# Relationship of Individual Athlete External Load, Session Rating of Perceived Exertion, and Athlete Playing Status Across a Collegiate Women’s Basketball Season

**DOI:** 10.3390/sports12120340

**Published:** 2024-12-06

**Authors:** Faith S. A. Brown, Jennifer B. Fields, Andrew R. Jagim, Erica L. King, Robert E. Baker, Angela Miller, Margaret T. Jones

**Affiliations:** 1 Frank Pettrone Center for Sports Performance, George Mason University, Fairfax, VA 22030, USA; fbrown20@gmu.edu (F.S.A.B.); jennifer.fields@uconn.edu (J.B.F.); jagim.andrew@mayo.edu (A.R.J.); eking20@gmu.edu (E.L.K.); 2 Sport, Recreation, and Tourism Management, George Mason University, Fairfax, VA 22030, USA; rbaker2@gmu.edu; 3 Department of Nutritional Sciences, University of Connecticut, Storrs, CT 06269, USA; 4 Sports Medicine, Mayo Clinic Health System, Onalaska, WI 54650, USA; 5 Biomedical Engineering, George Mason University, Fairfax, VA 22030, USA; 6 Research Methods and Educational Psychology, George Mason University, Fairfax, VA 22030, USA; amille35@gmu.edu

**Keywords:** basketball, external load, internal load, load monitoring

## Abstract

External (EL) and internal (IL) load are commonly used methods used to quantify training load in team sports. Playing time and playing position may influence the training loads for specific athletes throughout a season. The purpose of the current study was to evaluate the effect of athlete playing status and individual in-season practices on EL and IL across a collegiate women’s basketball season. Female basketball athletes were classified as high-minute (HMA; ≥15 min/game) or low-minute (LMA; <15 min/game) and wore microsensors during 53 practices for a total of 583 data points. EL was obtained via an inertial measurement unit (IMU) device that contained a triaxial accelerometer to obtain three-dimensional positioning data. IL and strength training (ST) load were determined via session rating of perceived exertion (sRPE) to create a daily summated value. Descriptive statistics indicate that athletes experienced individual differences in EL, ST, and IL throughout the season. A growth model showed that HMAs experienced higher EL than LMAs at the start of the season for practices (90.21 AU). Across all athletes, IL increased across the season (40.11 AU) and for each 1 unit of change in EL, IL increased by 1.04 AU. Repeated measures correlations identified a large relationship between IL and EL (r = 0.51, *p* < 0.001). A location-scale model indicated that the within-person variability of IL across all athletes was 3.29 AU but was not due to athlete playing status. It is recommended to base in-season training on individual loads and game demands to promote athlete readiness and improved sport performance.

## 1. Introduction

Monitoring individual athlete training loads in team sports is used to quantify physiological demands during training and competition [1,2]. Athlete training load data can inform programming decisions regarding the dosage, frequency, intensity, and volume of training loads, which take priority during the competitive season [3]. Appropriate load monitoring and management can also be used to reduce risks of injury, optimize recovery and athlete readiness, and improve sports performance, thus enhancing health and career longevity [4,5]. Monitoring external loads (ELs) provides insight into the physical volume and intensity demands placed on an athlete during training and competition. Examples of commonly monitored EL metrics include total distance [6], distance in various speed zones [7], acceleration/deceleration count [8], jumps [9], and proprietary metrics such as Playerload™ [10]. Playerload™ (PL) is a proprietary measure specific to Catapult units, is obtained through the use of a microsensor which contains triaxial accelerometer technology, and has been shown to be a valid and reliable EL measure in both outdoor and indoor sports [11,12,13].

Complementary to EL, internal load (IL) monitoring describes the psychophysiological response to the imposed external load demands [8]. While there are several ways to assess IL (e.g., heart rate zones, heart rate variability, biomarkers, subjective ratings of soreness and fatigue), session rating of perceived exertion (sRPE) is the most widely utilized measure in basketball athletes [14,15,16,17]. sRPE (sRPE = RPE × session duration) is an easy-to-implement, cost-effective, time-efficient, and valid measure of IL [14,15]. A dose–response relationship exists between EL and IL, but IL stress is highly individual and appears to be based on a variety of factors such as fitness level, playing status, and sport [18]. Therefore, quantifying both EL and IL is often recommended to provide a comprehensive understanding of an athlete’s adaptations to training, and to reduce the risk of injury, overtraining, and compromised performance [11].

The dose–response relationship between EL and IL is important to quantify for practical use. Quantifying the expected response for a certain “dose” of EL enables practitioners to have guidance when planning training target loads to elicit the desired IL response. Significant relationships between sRPE and EL metrics have been previously established in semi-professional men’s soccer [19], Australian football [20], collegiate women’s soccer [6], and semi-professional [8,21] and professional men’s basketball [22]. However, in basketball athletes, evidence of this relationship remains limited, particularly for women’s basketball. Currently, three studies [8,21,22] have explored the relationship between sRPE and EL metrics in men’s basketball athletes at the semi-professional and professional levels, with moderate-to-very large relationships existing between sRPE and various EL metrics (e.g., total deceleration count, total change of direction count, total acceleration count, total jump count, PL, total IMA count, high IMA count, and low IMA count). More research is warranted in collegiate women’s basketball athletes to determine if similar relationships exist to those observed for men’s basketball. To date, comparisons between the training loads of male and female basketball athletes have not been explored; however, differences in physiological, anatomical, neuromuscular, and biomechanical capabilities have been identified favoring male athletes [23]. These differences indicate that the training loads experienced, and their relationships may differ in female athletes. In addition, the individual variability of the EL and IL metrics has not been assessed in team sports. However, the need to adopt an individualized approach when monitoring training load in basketball athletes in regard to minute contribution has been suggested [24].

Additional factors have also been shown to influence athlete training loads in basketball throughout a season [25]. It has recently been shown that athlete training loads are differentiated by game minute contribution [26], playing level [27,28], and playing position [12,29]. Moreover, these factors were shown to influence ELs, resulting in athletes from the same team having differing IL responses [30]. Further, it has been suggested that IL responses will vary in athletes even when prescribed the same EL, due to differing physical characteristics and psychophysiological responses to exercise [18].

Despite athlete load monitoring and management being routinely implemented in collegiate athletic programs, there remains a lack of representation of published studies on female collegiate basketball athletes [31]. Therefore, the purpose of the current study was to evaluate the effect of athlete playing status and practices on individual practice EL and IL variability over a collegiate women’s basketball season.

## 2. Materials and Methods

### 2.1. Participants

National Collegiate Athletic Association Division I female basketball athletes from a single team (*n* = 11; mean ± SD; age 20.64 ± 1.63 years, height 175.77 ± 8.07 cm, body mass 71.38 ± 11.08 kg, body fat% 19.62 ± 7.53%) were classified into the following position groups: guard (*n* = 7), forward (*n* = 3), and center (*n* = 1). Guards included both point and shooting guards, which are backcourt athletes, playing primarily between the half-court line and 3-point line [32]. Forwards and centers are frontcourt athletes, playing primarily in the free-throw lane (i.e., the paint), and between the paint and 3-point line [32]. All basketball athletes were medically cleared for intercollegiate athletic participation by the University medical staff. The George Mason University Institutional Review Board for the use of human subjects in research approved the use of existing data under the determinant: Expedited Category 5 (Approval Date: 14 September 2023; IRB# 1674043-3).

### 2.2. Procedure and Instrumentation

#### 2.2.1. External Loads

The athletes (*n* = 11) wore microsensors [33] equipped with a 10 Hz GPS/GNSS and inertial measurement unit (IMU) technology (100 Hz triaxial accelerometer, gyroscope, and magnetometer; Optimeye S5, Catapult, Melbourne, Australia) for 53 in-season practices for a total of 583 data points. Units were turned on 15 min before use to allow time for adequate synchronization to the indoor receiver. To promote reliability, athletes wore the same unit throughout the season. These units have been previously shown to provide a valid measure of EL via PL [34]. Devices were worn according to manufacturer guidelines in a supportive harness positioned between the scapulae. After each practice, data were downloaded using the proprietary software, which automatically detected and filtered the data. Practices were live tracked on the laptop provided by the manufacturer. Data collection began with the team dynamic warm up and concluded at the end of the last drill. Data collection was paused between drills to avoid erroneous data collection.

The EL metric analyzed was PL, which is the sum of the forces from accelerations across all axes of movement, divided by 100 and reported in arbitrary units (AUs) [35].

$$\left(\sqrt{\frac{{\left(a_{y1}-a_{y-1}\right)}^{2}+{\left(a_{x1}-a_{x-1}\right)}^{2}+{\left(a_{z1}-a_{z-1}\right)}^{2}}{100}}\right)$$

PL was chosen as the EL metric because recent basketball research has utilized this metric to quantify EL and found moderate-to-strong relationships with IL [8,21].

#### 2.2.2. Session Rating of Perceived Exertion

RPE values were collected using the Borg CR-10 scale ~10 min post-practice and post-strength training sessions to avoid the recency effect. Individual responses were provided separately to avoid influencing others’ responses [36]. The sRPE was later calculated as the product of post-session RPE and session duration for each individual for practices (P-sRPE) and strength training sessions (ST load) [15]. Practice duration was quantified as beginning with the team dynamic warm up and concluding at the end of the last drill of the practice, not including stoppages of play.

Athlete playing status was classified as high-minute (HMA: ≥15 min/game) or low-minute (LMA: <15 min/game) and was determined for each game (*n* = 27). Athlete playing status was obtained via publicly available data. The 15 min threshold was established by the head coach. Depending upon the number of games played within a week, the practices preceding each game could range from 1 to 5. Athletes were labeled as HMAs or LMAs for each practice based upon game minutes played. The allocation of athletes as HMAs/LMAs was determined by the minutes played in the upcoming game and could vary for each game. This allocation did not follow a weekly format as 2–3 games may be played within one week. Since the HMA/LMA categorization was determined by each game, there was not a set number of athletes in either category across the entire season. The percentage of practices for each athlete classed as an HMA were calculated from 53 practices, which were developed and implemented by sport coaches. Practices occurred 4–5 times each week depending upon the game schedule. Practices consisted of a dynamic warm up, technical basketball skill work, basketball shooting drills, and tactical basketball strategy. Strength training sessions (*n* = 16; ST load) were developed and implemented by a National Strength and Conditioning Association Certified Strength and Conditioning Specialist (CSCS^®^) employed with George Mason University in Fairfax, Virginia, USA.

### 2.3. Statistical Analysis

Descriptives were calculated for individual P-sRPE, ST load, P-PL, and the percentage of practices out of the 53 total in which each athlete was categorized as an HMA (Table 1). A growth model examined P-sRPE across all practice timepoints with P-PL, practice timepoints, and athlete playing status as predictors. A location scale model was used to evaluate each athlete’s consistency of P-sRPE across all practice timepoints, using the practice timepoints and athlete playing status as predictors. Repeated measures correlations with 95% confidence intervals assessed the relationship of P-sRPE and P-PL. All analyses were conducted in RStudio (version 2023.12.1+402). Repeated measures correlations utilized the rmcorr package and the brms package was utilized for the location-scale model, which serves as a front end to the probabilistic programming language Stan (Stan Development Team, 2016) [37]. Statistical significance was set to *p* < 0.05 or the absence of 0 in 95% confidence intervals. Correlation coefficients (*r*) were classified: small (0.10–0.30), moderate (0.31–0.50), large (0.51–0.70), very large (0.71–0.90), or extremely large (0.91–1.00) [38].

## 3. Results

### 3.1. Athlete Playing Status and Practice

**Figure 1:**
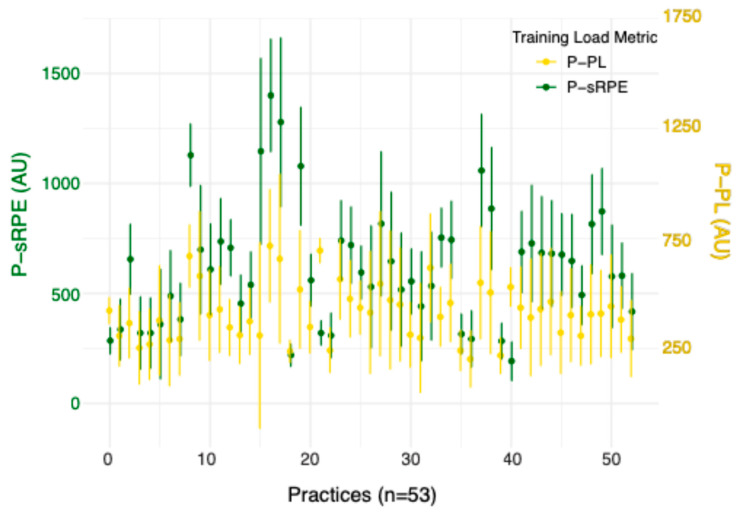
Practice sRPE (P-sRPE) and practice Playerload™ (P-PL) across all practice sessions and all participants.

Individual P-sRPE, ST load, P-PL, game minutes, and the percentage of practices out of 53 total practices that each athlete was categorized as an HMA are included in Table 1. P-sRPE, ST load, and P-PL grouped by athlete playing position are shown in Table 2. Figure 1 presents the average and standard deviations of P-sRPE and P-PL of 11 athletes across 53 practice sessions.

### 3.2. Growth Model

The growth model examined how the dependent variable (P-sRPE) changed across the season and how P-sRPE was affected by changes in P-PL. At the first practice, P-PL was 349.90 AU on average for all athletes and LMA athletes experienced an average P-PL of 90.21 AU less than HMAs (*p* = 0.005). On average, P-sRPE increased by 40.11 AU across athletes; however, the rate of growth diminished (−1.75 AU) over time (Figure 1). In addition, for each increase in P-PL, P-sRPE increased by 1.04 AU. A large relationship existed between P-sRPE and P-PL (r_rm_ = 0.51, *p* < 0.001) [95% CI: 0.43, 0.57].

### 3.3. Location-Scale Model

**Figure 2:**
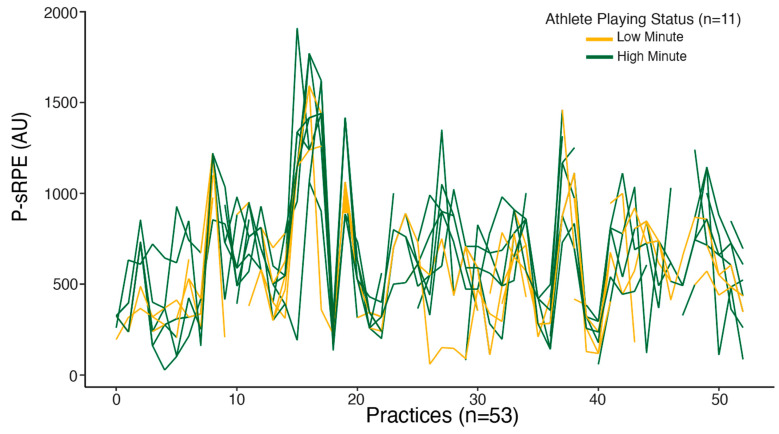
Athlete practice sRPE (P-sRPE) variability across all practice sessions.

Location-scale models were used to examine the between- and within-athlete variability in the intercept and variance across the repeated measures. P-PL and athlete playing status are predictors of P-sRPE. The average P-sPRE was 546.40 [95% CI: 481.00, 612.50] with a standard deviation between athletes of 28.20 [95% CI: 1.00, 78.81], and this was consistent across all practices. On average, HMAs were 74.90 [95% CI: 16.00, 132.40] higher than LMAs. As P-PL increased, so did P-sRPE, at a rate of 1.20 [95% CI: 1.00, 1.40]. The average within-person variability of P-sRPE for all athletes was 32.80 [95% CI: 31.30, 52.90]. There was considerable variation in P-sPRE across the season; however, this variability in P-sRPE did not significantly differ based on athlete playing status (0.40 [95% CI: −1.6, 2.1]). HMAs and LMAs were highly consistent with each other in their variation (Figure 2). Figure 2 includes individual sRPE across all 53 practices; the lines are color-coded by athlete playing status. This figure highlights the consistent variation all athletes experienced regardless of athlete playing status and the inconsistent within-person variability.

## 4. Discussion

The current study provides the first assessment of how practice EL and IL, along with the individual variability across a competitive basketball season, are affected by athlete playing status. The primary finding is that athletes may experience different EL and IL training load patterns across a competitive season despite playing on the same team. Additionally, the findings from the current study indicated that differences in practice EL are related to differences in athlete playing status and that strong relationships are present between PL and sRPE. Lastly, the within-person variability of PL is consistent across the athletes and is not affected by athlete playing status.

The descriptive data from this study suggest that position type has a more pronounced impact on training loads than athlete playing status. Our descriptives indicate that guards experience averages of 37.20 higher P-sRPE, 64.15 higher P-PL, and 11.87 lower ST load when compared to forwards (Table 2). In support, similar findings have been reported in professional men’s basketball, where guards accumulated greater sRPE weekly loads (2388 ± 1549) in training compared to forwards (1846 ± 1037) and experienced higher PL (326.30 ± 102.86) during practices than posts/forwards (284.26 ± 84.64) [26]. Our results indicated that when compared to guards, centers experienced 95.10 higher P-sRPE but a 41.20 lower ST load and 46.00 lower P-PL (Table 2). However, this may have been due to the limited sample size of centers (*n* = 1) in the current study.

The finding that athletes within the same team experienced different ELs is in alignment with previously documented research in male basketball athletes [26,29,30,32]. However, the research to support this in collegiate women’s basketball athletes is limited; therefore, the present study is the first to establish a profile of individual in-season practice loads in basketball athletes. In other research studies, profiles have been established of basketball athletes grouped by playing position, and differences in EL (e.g., total distance, high-speed running distance, accelerations, decelerations, PL, PL per minute, low and high IMA jumps, total jumps, explosive efforts, IMA acceleration, IMA deceleration, and change of direction (L/R)) based on playing position (i.e., guard, forward/post) [26,32]. In the present study, both EL and IL training loads varied by athlete (Table 1), and across playing positions (Table 2), with a high degree of individual variability.

The differences in training loads by position are likely influenced by the style of play and tactical strategies designed to combat a specific opponent and their playing style. A forward may be categorized as a frontcourt position [32] with the flexibility in skill to play near or far from the basket [39], which indicates that their loads may vary based on the tactical demands of their role in gameplay or practice. However, a recent study in professional men’s basketball reported that frontcourt athletes experience less total distance, accelerations, and decelerations but more high-speed running than backcourt athletes during gameplay [32]. In the current study, two guards experienced higher P-sRPE, ST load, and P-PL than the aforementioned forward athlete. The two guards were HMAs for ~93% of practices, but experienced higher loads than the forward, who was an HMA for 100% of practices, indicating that athletes of the same team can experience different P-sRPE, ST load, and P-PL across a competitive season. In Table 2, athletes are grouped by position and the average P-PL values for all guards are higher than all forwards, but the P-sRPE and ST load for forwards are higher than guards. This difference in examining loads by position group highlights the need for individual analysis, as collapsing all athletes together as a team for analysis may lead to inaccurate conclusions regarding athlete demands and training load accumulation or patterns throughout the season.

We sought to examine the effect of practices and playing status across a basketball season on EL and IL using growth modeling, which has yet to be assessed in this population. HMAs experienced higher P-PL than LMAs, and across the season P-sRPE gradually increased for all athletes, but growth slowed as the season progressed. The increase in sRPE over time may have been a result of cumulative fatigue, and the decrease in the growth of sRPE may have occurred due to athlete adaptation to the imposed demands of the competitive phase. As a result, there could be a dose–response relationship that is modeled by the relationships of P-sRPE and P-PL in the current study, with longitudinal trends indicating an accumulated state of fatigue if sRPE drifts upwards, despite stable session durations. When examining Figure 2, a large spike is present in sRPE and P-PL from practices 15–20. This peak occurred during the time when the basketball athletes had a mandated break in competition for final exams. An increase in load of no more than 10% per week is recommended to elicit the desired adaptations [40,41]. When considering that athletes were not under game loads two to three times a week during this period, the increase in practice loads likely balanced out their weekly load. To further support this increase in training loads, research in elite athletes supports the use of chronic high loads to buffer athletes from injury [41], provided that these loads are approached gradually over time. P-PL and sRPE following practice 20 and beyond indicate that practice volume was reduced to what was seen earlier in the season, and athletes subsequently re-adapted to the in-season demands.

It could be hypothesized that fatigue accumulation across the season may be of heightened concern for HMAs compared to LMAs. Recent research into collegiate women’s basketball has shown that HMAs experience higher game loads but lower practice loads than LMAs [26]. However, the results from this study yield conflicting results which can be observed in Table 1, where the descriptive data of individual athlete loads and the percentage of time they were categorized as an HMA are presented. In addition, the growth model indicated that HMAs experienced a higher PL by 90.21 AU at the first practice of the season. HMAs may experience higher practice training loads than LMAs, especially in-season, as they are the athletes who must thoroughly practice the tactical strategies for an upcoming game and thus may spend more time than LMAs obtaining reps. However, during practices, it is logical to expose LMAs to training with a heightened emphasis on skill acquisition, technical development, and maintaining fitness levels, which may lead to higher EL and subsequently a higher sRPE than HMAs. This approach would support the concept of individualizing training loads for specific athletes throughout a season. As depicted in Table 1, individual loads varied, indicating that athletes experienced training differently, which may have been a result of differing physiological responses and individual adjustments to sport practices and ST sessions.

The large relationship observed from the repeated measure correlation between P-PL and sRPE indicates a dose–response relationship. As such, the IL response can be anticipated based on the EL dose. Therefore, those utilizing EL without IL can have increased awareness of expected IL responses. In addition, this large relationship is in support of previous research in collegiate female soccer athletes [6], semi-professional male basketball athletes [8,21], and professional male basketball athletes [22]. In the present study, EL was quantified by PL, and IL was quantified by sRPE utilizing the Borg CR-10 scale. In semi-professional men’s basketball athletes, significant moderate relationships were reported between EL and sRPE during training [8,21]. In professional women’s basketball, a moderate relationship between sRPE and PL per minute was found, but the relationship of PL and sRPE was not examined [42]. However, it is logical to presume that the relationship of PL and sRPE would be similar as PL per minute is an intensity metric derived from the original PL calculation [43]. To date, the relationship between sRPE and EL metrics has not been explored in collegiate women’s basketball. Further, as mentioned previously, basketball research typically evaluates athlete group loads such as the entire team during a specific time of the season [44], game minute contribution [26], playing level [27,28], and playing position [12,29,30,32].

In the location-scale model presented in this study, although the within-person variability was not consistent, it was not due to athlete playing status as hypothesized. This may be explained by the lack of a consistent group of five to seven athletes who achieved constant HMA status. Out of the 11 athletes, only 1 athlete was an HMA for all 27 games across the season. While the reason for the high degree of variability was unable to be determined based upon the variables included in the analysis, it was consistent across the entire team. The average variability was 0.09 across all athletes, but the intra-athlete variability was 0.20 along with narrow confidence intervals. Thus, it can be assumed that all athletes experienced the same phenomenon impacting their consistency across the season, and it was likely not a result of individual differences in load during training or games.

Further research that explores the demands of collegiate female basketball athletes remains warranted [31]. As demonstrated in the current study, assessing the variability of individual athletes may yield more insightful results than what is obtained by grouping athletes. The analysis presented in the current study may be used as a guideline for analyzing training loads in future research.

## 5. Limitations

The current study is not without limitations. EL and IL obtained during practice sessions were examined. Certainly, athletes have other demands placed upon them that are not represented by measures of EL and IL. Examining EL metrics other than PL may result in different conclusions. Further, a singular team was utilized for this study, which has certain practice tendencies and characteristics that may differ from other teams. Due to the nature of basketball rosters, and the inclusion criteria for this study, the samples by position were small. However, this was mitigated by utilizing a repeated measures design in the statistical analysis. Despite these limitations, our findings offer valuable insight into the individual demands and variance that D-I WBB athletes experience during training and the relationship between EL and IL.

## 6. Conclusions

The main findings from the current study indicate that collegiate female basketball athletes of the same team experience different training loads across a competitive season. The differing practice ELs are due to differences in athlete playing status at the start of the season. Large relationships are present between EL and IL, and the within-person variability of sRPE was inconsistent across the season for all athletes in a similar fashion but was not due to athlete playing status.

## Figures and Tables

**Table 1:** Training loads for individual athletes across in-season practices and strength training sessions.

Position	Game Minutes	% of Practices HMA	P-sRPE (AU) (*n* = 53)	ST Load (AU) (*n* = 16)	P-PL (AU) (*n* = 53)
Forward	34.11 ± 6.16	100	641.8 ± 342.0	146.4 ± 60.2	430.2 ± 177.4
Guard	27.89 ± 6.51	94	649.0 ± 350.3	184.6 ± 93.4	493.8 ± 167.9
Guard	25.30 ± 8.86	92	696.8 ± 381.8	193.9 ± 62.5	476.4 ± 183.0
Forward	20.44 ± 9.64	87	613.0 ± 309.6	201.8 ± 65.7	287.9 ± 112.2
Guard	29.07 ± 9.64	87	588.9 ± 313.0	156.3 ± 47.3	401.2 ± 142.6
Center	17.30 ± 8.93	79	704.8 ± 367.6	123.6 ± 59.3	386.0 ± 144.4
Guard	14.78 ± 10.68	72	617.9 ± 315.8	141.8 ± 59.8	440.5 ± 149.8
Guard	18.78 ± 11.17	58	613.6 ± 344.9	168.1 ± 62.5	339.6 ± 135.6
Guard	4.41 ± 5.93	4	569.0 ± 281.1	149.3 ± 40.6	482.9 ± 172.6
Forward	2.63 ± 3.87	0	610.9 ± 355.4	201.7 ± 53.9	346.5 ± 127.0
Guard	0.30 ± 1.07	0	499.5 ± 367.2	165.0 ± 53.1	375.9 ± 151.7

Values are mean *±* SD; AU = arbitrary unit; P-sRPE = practice session rating of perceived exertion (AU); P-PL = practice Playerload™ (AU); HMA = high-minute athlete.

**Table 2:** Athlete in-season practice loads by playing position.

Position	P-sRPE (AU) (*n* = 53)	ST Load (AU) (*n* = 16)	P-PL (AU) (*n* = 53)
Guard (*n* = 7)	609.7 ± 337.6	164.8 ± 61.6	432.0 ± 166.9
Forward (*n* = 3)	646.9 ± 345.9	176.67 ± 73.2	367.85 ± 152.1
Center (*n* = 1)	704.8 ± 367.6	123.6 ± 59.3	386.0 ± 144.4

Values are mean *±* SD; AU = arbitrary unit; P-sRPE = practice session rating of perceived exertion (AU); P-PL = practice Playerload™ (AU).

## Data Availability

Data are contained within the article.
